# Deep Learning-Based Molecular Fingerprint Prediction for Metabolite Annotation

**DOI:** 10.3390/metabo15020132

**Published:** 2025-02-14

**Authors:** Hoi Yan Katharine Chau, Xinran Zhang, Habtom W. Ressom

**Affiliations:** Department of Oncology, Lombardi Comprehensive Cancer Center, Georgetown University Medical Center, Washington, DC 20057, USA; hc820@georgetown.edu (H.Y.K.C.); xz562@georgetown.edu (X.Z.)

**Keywords:** deep learning, molecular fingerprint prediction, metabolite identification, LC-MS/MS

## Abstract

**Background/Objectives:** Liquid chromatography coupled with mass spectrometry (LC-MS) is a commonly used platform for many metabolomics studies. However, metabolite annotation has been a major bottleneck in these studies in part due to the limited publicly available spectral libraries, which consist of tandem mass spectrometry (MS/MS) data acquired from just a fraction of known compounds. Application of deep learning methods is increasingly reported as an alternative to spectral matching due to their ability to map complex relationships between molecular fingerprints and mass spectrometric measurements. The objectives of this study are to investigate deep learning methods for molecular fingerprint based on MS/MS spectra and to rank putative metabolite IDs according to similarity of their known and predicted molecular fingerprints. **Methods**: We trained three types of deep learning methods to model the relationships between molecular fingerprints and MS/MS spectra. Prior to training, various data processing steps, including scaling, binning, and filtering, were performed on MS/MS spectra obtained from National Institute of Standards and Technology (NIST), MassBank of North America (MoNA), and Human Metabolome Database (HMDB). Furthermore, selection of the most relevant *m*/*z* bins and molecular fingerprints was conducted. The trained deep learning models were evaluated on ranking putative metabolite IDs obtained from a compound database for the challenges in Critical Assessment of Small Molecule Identification (CASMI) 2016, CASMI 2017, and CASMI 2022 benchmark datasets. **Results**: Feature selection methods effectively reduced redundant molecular and spectral features prior to model training. Deep learning methods trained with the truncated features have shown comparable performances against CSI:FingerID on ranking putative metabolite IDs. **Conclusion**: The results demonstrate a promising potential of deep learning methods for metabolite annotation.

## 1. Introduction

One of the most commonly used methods for metabolite annotation is spectral matching of tandem mass spectrometry (MS/MS) data against reference MS/MS data in spectral libraries. Based on the similarity of the spectrum pair, a score is calculated for each putative metabolite ID if the corresponding MS/MS spectrum is present in the spectral library [1,2]. This type of annotation, however, is highly limited by the size of reference compounds the spectral libraries represent [3,4]. For example, while PubChem currently consists of over 119 million unique chemical structures, the NIST23 tandem mass spectral library has reference spectra for 51,501 unique compounds [5,6,7]. This shows the difficulty of annotating a very large number of analytes for which no matching reference spectra are available, needless to say the immense challenge of annotating compounds that have not yet been included in the compound databases [8,9]. Methods that can accurately rank putative metabolite IDs based on mass spectrometric measurements are highly desired as an alternative to spectral matching.

There are many ongoing studies to address the prevailing challenges in metabolite annotation. Some of these studies focus on predicting molecular formulae. For example, BUDDY is a bottom-up method to predict molecular formulae. It uses the query MS/MS spectrum to create a set of fragment-to-mass loss and formula candidates, then matches the predicted information to a mass spectrometry (MS) spectrum and precursor *m*/*z* value to score the predicted formula [10]. Metabolite Inference with Spectrum Transformers for Chemical Formula prediction (MIST-CF) uses transformers to predict molecular formulae based on MS and MS/MS spectra [11]. SIRIUS calculates all possible molecular formulae based on precursor *m*/*z* and applies MS and MS/MS data to validate the possibility of the predicted formulae by computing fragmentation trees using support vector machines (SVMs) [12,13,14]. A similar de novo fragmentation tree construction approach with a probabilistic model is applied by Competitive Fragmentation Modeling (CFM) for metabolite annotation using MS/MS spectra [15,16]. Other studies focus on predicting compound structures. For example, MSNovelist predicts compound structures on the basis of Simplified Molecular Input Line Entry System (SMILES) using recurrent neural networks (RNN) [17]. CSI:FingerID and IDSL_MINT predict molecular fingerprints using SVMs or transformers, respectively [18,19]. CSI:FingerID has also developed a deep kernel learning method for fingerprint prediction [20]. METASPACE-ML performs metabolite annotation through MS images using a decision tree model [21]. 

Previously we evaluated various models and developed a metabolite annotation tool, MetFID, using convolutional neural network (CNN) for molecular fingerprint prediction based on MS/MS spectra [22,23,24]. The promising potential of CNN ignited our interest in exploring other deep learning methods. 

In this paper, we compare the performances of three deep learning models in molecular fingerprint prediction based on MS/MS spectra. Briefly, putative metabolite IDs (referred as candidates in later text) were obtained from a compound database based on precursor *m*/*z* values of the unknown MS/MS spectra. These candidates were ranked according to similarity of their molecular fingerprints to those predicted by the deep learning models. To further improve the ranking performance, the molecular fingerprint similarity scores were combined with formula prediction scores obtained from SIRIUS. Training MS/MS spectra acquired in either positive mode and/or negative mode from tens of thousands of compounds were obtained from NIST23, MoNA, and HMDB [5,6,25,26]. The molecular fingerprint for each compound was calculated using PyFingerprint and OpenBabel [27] based on information obtained from predefined structure libraries such as FP3, FP4, PubChem, Molecular ACCess System (MACCS), and Klekota-Roth. Following MS/MS data processing and selection of the most relevant MS/MS features and molecular fingerprints, three deep learning models, a deep neural network (DNN), a CNN, and an RNN in long short-term memory (LSTM) architecture (referred as RNN in later text), were trained. The trained models were then used to predict the molecular fingerprints for the challenges in the CASMI 2016, CASMI 2017, and CASMI 2022 benchmark datasets [28,29,30,31]. To ensure structure-disjoint evaluation, all MS/MS spectra whose first parts of InChIKey overlap with the challenges in any of the three benchmark datasets were excluded from the training set. For each challenge, candidates were retrieved from a compound database based on the challenge’s precursor *m*/*z* value. The candidates were ranked on the basis of similarity of their molecular fingerprints to those predicted by the deep learning methods as well as a molecular formula prediction score obtained from SIRIUS. Finally, top-*k* ranking results were calculated to evaluate the performances of the deep learning methods and compare them with CSI:FingerID. The results show deep learning models offer great potential to address the metabolite annotation challenge.

## 2. Materials and Methods

### 2.1. Overview

**Figure 1:**
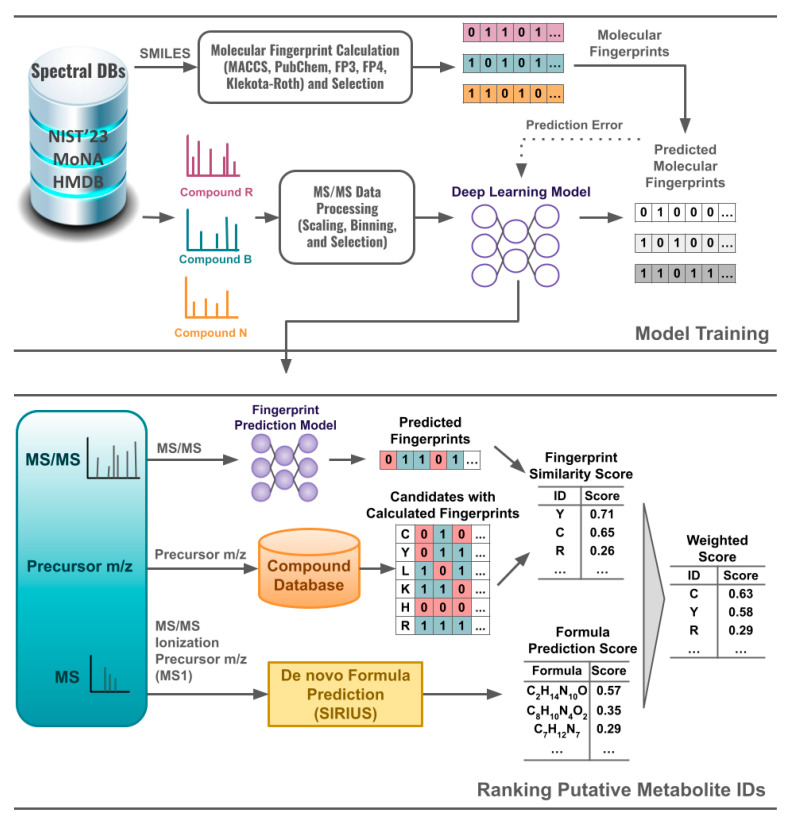
Workflow of a deep learning-based metabolite annotation that includes MS/MS data processing, feature selection, model training, molecular fingerprint prediction, molecular formula prediction, candidate retrieval, and candidate ranking.

Figure 1 illustrates overall workflow for deep learning-based model training and for ranking putative metabolite IDs using the trained model. The workflow includes the following steps: MS/MS data processing, molecular fingerprint calculation, feature selection, deep learning model training, molecular fingerprint prediction, molecular formula prediction, candidate retrieval, and candidate ranking.

### 2.2. MS/MS Data Processing

We processed LC-MS/MS spectra obtained from NIST23, MoNA, and HMDB [5,6,25,26]. Each spectrum was annotated with reference compound information and instrument settings including metabolite ID, molecular formula, InChIKey, SMILES, precursor *m*/*z*, adduct, ionization mode, collision energy, etc.

Data processing steps included peak intensity scaling, binning, and filtering bins based on frequency of occurrence across spectra. Specifically, we scaled the peaks of each spectrum to have their relative intensities range between 0 and 100. We then separated the spectra into two based on their ionization mode (positive and negative). We filtered out spectra that have no or more than one precursor mass and spectra that have less than five peaks. These steps resulted in 1,005,931 positive-mode spectra representing 38,339 compounds and 264,153 negative-mode spectra representing 18,213 compounds. Combined, these MS/MS spectra represent 43,386 compounds. 

Prior to binning, we removed peaks in each spectrum that fell outside the mass range between 100 and 1010 Dalton and peaks whose scaled intensity values were less than 1. For each spectrum, the top 20 peaks with the highest relative intensities were selected. These peaks were mapped into bins of 0.01 Dalton size. Within each bin, the intensity values were summed to produce a binned intensity vector. This step resulted in 91,001 bins. Bins that had intensity values coming from less than 0.1% of the training spectra were removed. This filtering step was performed separately for positive and negative mode spectra, resulting in 2010 bins in the positive mode and 2015 in the negative mode.

Table 1 presents the number of MS/MS spectra and compounds we used for training deep learning models. In total, 1,270,084 MS/MS spectra representing 43,386 compounds were used for training.

To evaluate the performances of the trained models in ranking putative metabolite IDs, we considered three benchmark datasets from the Critical Assessment of Small Molecule Identification (CASMI), which is an open contest organized by scientists for testing the capability of computational compound identification methods and tools [28,29,30,31]. So far, the contest has been held for 6 years, and each year, a set of MS/MS and MS spectral benchmark data is created for testing the computational methods. As shown in Table 1, we used three datasets, CASMI 2016, CASMI 2017, and CASMI 2022, consisting of 951 challenges and representing 857 compounds to evaluate the deep learning models trained in this study. The training and testing MS/MS spectra represent distinct compounds to ensure a structure-disjoint evaluation.

### 2.3. Molecular Fingerprint Calculation

Molecular fingerprints for all compounds in the training and testing datasets were calculated based on their SMILES string using PyFingerprint “available from http://github.com/hcji/Pyfingerprint (accessed on 22 Jul 2024)” and OpenBabel [27]. The fingerprints obtained from predefined structure libraries including FP3, FP4, PubChem, MACCS, and Klekota-Roth were transformed into vectors of binary entries, resulting in 6269 binary vectors [7,32]. We filtered out fingerprints that appear as 1 or 0 across all compounds in the training set, as these represent properties shared by all or none, respectively, of the compounds. Additionally, redundant fingerprint vectors, i.e., vectors that have the same binary entries across all compounds, were condensed into one vector. These steps reduced the size of the fingerprint vector to 4606. 

### 2.4. Feature Selection

A supervised method was used to select the most relevant *m*/*z* bins and molecular fingerprints prior to model training. For bin selection, we started with 2010 in the positive mode and 2015 bins in the negative mode. We then applied multi-layer perceptron (MLP) models to map the relationships between these bins and 4606 molecular fingerprints. Based on the F1 score of each bin in a 5-fold cross-validation, the top performing 500 bins were selected for the positive and negative mode training sets separately.

For fingerprint selection, CNN models were trained using the top 500 bins selected in the previous step. Through a 5-fold cross-validation, F1 scores were calculated for each of the 4606 fingerprints. Fingerprints with an F1 score of over 0.8 were selected for subsequent deep learning model training. Thus, 192 fingerprints were selected for the positive-mode data and 272 fingerprints for the negative-mode training sets. Table 2 shows the number of features obtained following filtering and supervised feature selection. Both MLP and CNN models were constructed using the Keras module from the TensorFlow Python package [33]. The hyperparameters of these models are described in Supplementary Material S1.

### 2.5. Deep Learning Model for Molecular Fingerprint Prediction

**Figure 2:**
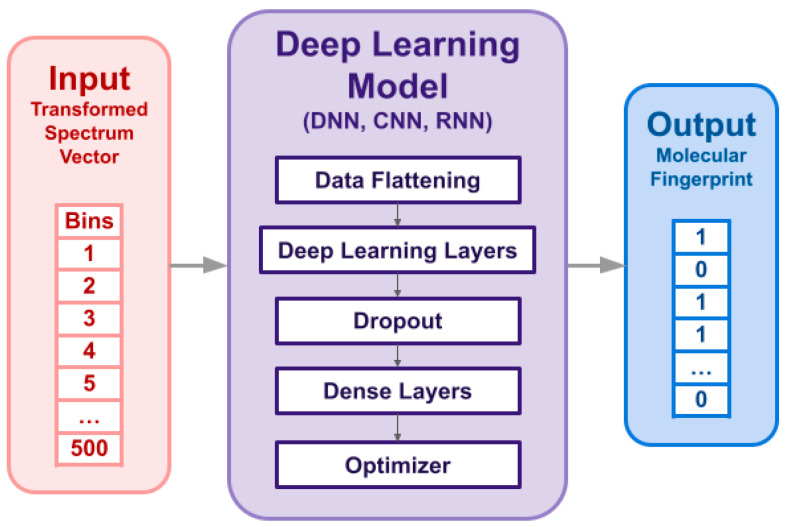
Architecture of a deep learning model for predicting molecular fingerprints based on MS/MS spectra transformed into vectors.

We considered DNN, CNN, and RNN for fingerprint prediction, extending on our previous evaluation of multiple machine learning and deep learning methods [22,23,24]. The models were trained using the Keras module from the TensorFlow Python package [33]. A general deep learning model architecture is shown in Figure 2. Please refer to Supplementary Material S2 for the hyperparameters of each model. We trained separate models for the positive and negative mode data.

During the training process, the model adjusts its parameters based on the Tanimoto similarity score (Equation (1)) between the predicted and true fingerprints. In the equation, TN, TP, FN, and FP represent the number of true negatives, true positives, false negatives, and false positives, respectively.

(1) $$\mathrm{Tanimoto}\;\mathrm{Similarity}\;\mathrm{Score}=\frac{TP}{FP+FN-TP}$$

When testing the model, an MS/MS spectrum acquired from an unknown metabolite was transformed into a vector of pre-specified bins. The vector was used as an input to the pre-trained deep learning model to predict a molecular fingerprint. Meanwhile, the precursor *m*/*z* value of the MS/MS spectrum was used to search through a compound database to obtain metabolite candidates. For each candidate, a Tanimoto similarity score was calculated by comparing the predicted fingerprint with the candidate’s fingerprint. Thus, candidate ranking was performed for each query spectrum based on the Tanimoto similarity scores.

When we compared the fingerprint prediction performance across the three deep learning models, we cross-validated the models with training data and calculated Tanimoto similarity score (Equation (1)), F1 score (Equation (2)), and mean Matthews correlation coefficient (MCC) score (Equation (3)).

(2) $$F1\;\mathrm{score}=\frac{2\times Precision\times Recall}{Precision+Recall}$$

where Precision = $\frac{TP}{TP+FP}$ Recall = $\frac{TP}{TP+FN}$.

(3) $$\mathrm{MCC}\;\mathrm{score}=\frac{TP\times TN-FP\times FN}{\sqrt{\left(TP+FP\right)\times \left(TP+FN\right)\times \left(TN+FP\right)\times (TN+FN)}}$$

### 2.6. Molecular Formula Prediction

We used SIRIUS to predict molecular formulae for each CASMI challenge based on MS and MS/MS spectra. The molecular formula prediction and fingerprint prediction scores were weighted and linearly combined to improve the accuracy of ranking metabolite candidates. SIRIUS calculates all the possible formulae based on precursor *m*/*z* value, MS, and MS/MS to narrow down the candidates by building fragmentation trees and scoring the candidates [14]. Min–max normalization was applied to the SIRIUS formula prediction score from all candidates to ensure the candidate scores were on a similar scale for fair comparison. The *m*/*z* tolerance was set to 5 ppm to provide de novo formula predictions. SIRIUS ranked the candidates based on a SIRIUS score that is a combination of isotope score and fragmentation tree score. 

### 2.7. Performance Evaluation

We used the CASMI 2016, CASMI 2017, and CASMI 2022 benchmark datasets to evaluate performance of the trained deep learning models and compare their performances against CSI:FingerID. The benchmark datasets consist of 578 challenges in positive mode and 373 challenges in negative mode. Table 1 presents the number of spectra and compounds in each benchmark dataset. The compounds in the training and testing datasets were made distinct by removing compounds that share the same first 14 characters of InChIKey from the training set to enable structure-disjoint evaluation.

We retrieved candidate metabolites for each challenge in the benchmark datasets based on precursor *m*/*z* values within 5 ppm tolerance from a compound database comprising more than 1.7 million compounds. The positive-mode spectra are assumed to have [M + H]^+^, [M + NH4]^+^, or [M + Na]^+^ as their adducts. The adducts for the negative mode spectra are assumed to be [M + Cl]^−^, [M + FA − H]^−^, or [M − H]^−^. The fingerprint similarity score and formula prediction (i.e., SIRIUS) score were combined into an overall score for each challenge by reweighing the two scores in a 7:3 ratio. The compound candidates were then re-ranked according to the overall score. 

When considering whether a candidate is a hit, it must share the first part of InChIKey with the true candidate provided by the CASMI contest. After finding all hits in the final ranking result, based on the rankings of each of the benchmark datasets, a top-*k* ranking of true candidates was calculated. The performance of a model was evaluated based on the top-*k* rankings for the combined positive mode and negative mode challenges.

## 3. Results and Discussions

### 3.1. Evaluation of Molecular Fingerprint Prediction Models via Cross-Validation

We compared the fingerprint prediction performances among the three deep learning models. Table 3 and Table 4 show the 5-fold cross-validation performance of the models trained separately for the positive and negative modes.

We previously published results obtained by comparing various models such as logistic regression, MLP, and SVM with CNN [23]. Among these, we found CNN’s performance in molecular fingerprint prediction was the best. In the present study, we focused on three deep learning models using more recent MS/MS spectra for training and additional benchmark datasets for performance evaluation. Furthermore, we improved the MS/MS data processing approach and performed supervised methods to select the most relevant *m*/*z* bins and fingerprints. The latter helped reduce the number of *m*/*z* bins and molecular fingerprints while retaining a comparable if not better fingerprint prediction power. This shows that a fraction of the training features is adequate to map the spectral-molecular fingerprint relationships. We observed that including an excessive number of insignificant features may have a negative impact depending on the type of model used.

Selecting suitable performance metrics is challenging. As shown in Table 3 and Table 4, we used F1 score, Tanimoto similarity score, and MCC score to evaluate each model’s performance in molecular fingerprint prediction by cross-validation. During the cross-validation, the CNN model obtained the best F1, Tanimoto similarity, and MCC scores. However, as illustrated in the next section, in evaluating the top-*k* ranking performance using benchmark datasets, the RNN model performed the best. This indicates the top-*k* ranking results cannot be directly inferred from the metrics we used to evaluate molecular fingerprint prediction by cross-validation.

### 3.2. Top-k Ranking Performance Evaluation of Deep Learning Models via CASMI Benchmark Datasets 

To evaluate the benefit of the deep learning models in ranking candidates, we randomly listed the candidates obtained for each benchmark challenge 100 times and obtained the average top-*k* ranking results. For this evaluation, we considered formula unknown and formula known cases. The random ranking results for the three benchmark datasets are shown in Table 5, Table 6 and Table 7 under the columns marked as “Random”. The formula predicted case was not considered here since the MS/MS data were not used—only the candidates were randomly listed for evaluation. Also presented in these tables are the top-*k* ranking results we obtained for each CASMI benchmark dataset using the trained deep learning models. As illustrated in the tables, all three deep learning models performed far better than random ranking of candidates in both cases. 

Assuming the molecular formula is unknown (formula unknown case), the top-*k* ranking performance of RNN surpassed DNN and CNN when tested with the three CASMI benchmark datasets. 

We used SIRIUS to predict molecular formulae for the benchmark datasets and integrated the formula prediction scores with the fingerprint scores for ranking candidates. For some CASMI challenges, the true formula was not in the candidate list generated by SIRIUS. Thus, only fingerprint prediction scores were used for these candidates. Table S1 shows the SIRIUS’s formula prediction accuracy for the challenges in the three benchmark datasets. When we introduced formula prediction results from SIRIUS (formula predicted case), RNN remained the best in top-*1* ranking of the challenges in the CASMI 2016 and CASMI 2022 datasets, and CNN performed the best in ranking the challenges in CASMI 2017. When we considered a shorter list of candidates assuming the formula is known (formula known case), RNN performed the best for the CASMI 2016 and CASMI 2022 datasets, while CNN was the best for the CASMI 2017 dataset. While the top-*k* ranking presented in Table 5, Table 6 and Table 7 were obtained by combining the results from the positive and negative mode models, the ranking performances for the positive and negative mode models are separately shown in Supplementary Materials Tables S2–S7.

### 3.3. Comparison of Deep Learning Models with CSI:FingerID

Here, we compare the performances of the three deep learning models (DNN, CNN, and RNN) against CSI:FingerID considering formula predicted and formula known cases. This is because CSI:FingerID uses, by default, formula prediction results from SIRIUS in addition to fingerprint prediction for ranking metabolite candidates [12,13,14,18]. It also gives users the option to provide a specific formula for each challenge. While MS, MS/MS, and precursor mass *m*/*z* values were available for each challenge in CASMI 2016, all except 45 challenges were missing MS data in CASMI 2017. For CASMI 2022, only MS/MS and precursor *m*/*z* values were used. 

In our evaluation using the CASMI 2016 dataset, CSI:FingerID performed better than all three deep learning models in both formula predicted and formula known cases. When testing with the CASMI 2017 dataset considering the formula predicted case, both CNN and RNN performed better than CSI:FingerID. However, CNN and RNN had a performance similar to CSI:FingerID in the formula known case. For the CASMI 2022 dataset, all three deep learning models significantly outperformed CSI:FingerID in both cases.

For the training data used in this study, we ensured that the compounds represented by the training MS/MS spectra were distinct from those represented by the benchmark datasets so that the model performances were evaluated based on previously unseen datasets. However, we found a few of the CASMI challenges in CSI:FingerID’s training set. Hence, we are unable to confirm structure-disjoint evaluation of the CASMI challenges using CSI:FingerID.

In summary, this study demonstrates the promising potential of CNN and RNN for accurate fingerprint prediction. Combining fingerprint prediction with formula prediction scores leads to a significant improvement in ranking putative metabolite IDs. Our future work will focus on further optimizing deep learning models including CNN and RNN as well as exploring other architectures, such as graph neural networks (GNN) and graph transformers, that have been reported to perform very well in modeling complex relationships to perform more accurate ranking of putative metabolite IDs, thereby addressing the challenges in metabolite annotation.

## 4. Conclusions

In this paper, we present three deep learning models (DNN, CNN, and RNN) trained to predict molecular fingerprints based on MS/MS spectra. The performance of the models in ranking putative metabolite IDs (candidates) was evaluated via three (CASMI 2016, CASMI 2017, and CASMI 2022) benchmark datasets. The candidates were obtained from a compound database based on the precursor *m*/*z* of the challenges in the benchmark datasets. For each challenge, we considered three cases: a list of candidates assuming the formula is known, the same list with formula prediction scores obtained from SIRIUS, and a shorter list assuming the formula is known. The top-*k* ranking results obtained from the latter two cases indicate that CNN and RNN performed slightly better than CSI:FingerID.

Our future work will focus on optimizing the CNN and RNN and other models, such as graph neural networks and graph transformers, for both molecular fingerprint and formula prediction based on MS/MS and MS data. We will also expand our selection of molecular fingerprints by including hashed fingerprints, potentially adding more molecular structures to improve prediction accuracy. To prevent potential overestimation of model performance during evaluation, we will explore additional criteria to ensure the compounds represented by the training and testing spectra are more distinct. This will be accomplished by making sure that compounds that are likely to have similar fragmentation patterns do not appear in both the training and testing datasets based on their InChIKeys, SMILES, molecular fingerprints, etc.

Finally, we will investigate optimization methods such as grid search and Bayesian optimization via cross-validation to determine the best coefficients that combine the scores from molecular fingerprint and formula prediction. We believe combining these two scores has a promising potential for improving the performance of ranking putative metabolite IDs obtained by mere precursor *m*/*z* values, thereby addressing the challenge in metabolite annotation.

## Figures and Tables

**Table 1:** Number of MS/MS spectra and compounds used for training and testing datasets deep learning models.

Data	Source(s)	Number of MS/MS Spectra		Number of Compounds		
		Positive	Negative	Total	Positive	Negative
Training data	MoNA, NIST 23, HMDB	1,005,931	264,153	43,386	38,339	18,213
Testing data	CASMI 2016	127	81	188	127	81
	CASMI 2017	147	96	169	147	96
	CASMI 2022	304	196	500	304	196

**Table 2:** Number of *m*/*z* bins and molecular fingerprints before and after filtering and supervised feature selection.

Data	Sources	Original Number		After Filtering		After Supervised Selection	
		Positive	Negative	Positive	Negative	Positive	Negative
Bins	MoNA, NIST 23, HMDB	91,001		2010	2015	500	500
Fingerprints	PubChem, MACCS, Klekota-Roth, FP3, FP4	6269		4606		192	272

**Table 3:** Cross-validation F1 score, Tanimoto score, and mean Matthews correlation coefficient (MCC) score of DNN, CNN, and RNN with positive-mode data [34].

Positive Mode	DNN	CNN	RNN
F1	50%	71%	51%
Tanimoto	34%	55%	35%
MCC	51%	71%	51%

**Table 4:** Cross-validation F1 score, Tanimoto score, and mean Matthews correlation coefficient (MCC) score of DNN, CNN, and RNN with negative-mode data.

Negative Mode	DNN	CNN	RNN
F1	56%	79%	56%
Tanimoto	39%	65%	39%
MCC	56%	78%	57%

**Table 5:** Top-*k* ranking performance of randomly shuffled candidate list, prediction by deep learning models, and prediction by CSI:FingerID on CASMI 2016 benchmark dataset. The best result in each of the testing conditions is indicated in bold font.

Method	Formula Unknown				Formula Predicted				Formula Known				
	Random	DNN	CNN	RNN	DNN	CNN	RNN	CSI:FingerID	Random	DNN	CNN	RNN	CSI:FingerID
Top-*1*	2%	10%	23%	**27%**	27%	25%	28%	**48%**	14%	42%	46%	47%	**54%**
Top-*3*	9%	19%	29%	**32%**	45%	35%	33%	**59%**	27%	60%	60%	61%	**69%**
Top-*5*	15%	27%	34%	**36%**	52%	40%	41%	**60%**	32%	65%	67%	67%	**72%**
Top-*10*	27%	40%	45%	**47%**	62%	54%	54%	**63%**	37%	73%	77%	**78%**	75%

**Table 6:** Top-*k* ranking performance of randomly shuffled candidate list, prediction by deep learning models, and prediction by CSI:FingerID on CASMI 2017 benchmark dataset. The best result in each of the testing conditions is indicated in bold font.

Method	Formula Unknown				Formula Predicted				Formula Known				
	Random	DNN	CNN	RNN	DNN	CNN	RNN	CSI:FingerID	Random	DNN	CNN	RNN	CSI:FingerID
Top-*1*	3%	8%	16%	**28%**	9%	**33%**	26%	14%	6%	20%	**38%**	33%	37%
Top-*3*	11%	11%	23%	**28%**	16%	**34%**	28%	20%	16%	27%	46%	46%	**48%**
Top-*5*	16%	13%	26%	**30%**	19%	**47%**	31%	21%	25%	37%	50%	44%	**52%**
Top-*10*	28%	21%	30%	**37%**	27%	**50%**	37%	22%	38%	44%	**56%**	52%	**56%**

**Table 7:** Top-*k* ranking performance of randomly shuffled candidate list, prediction by deep learning models, and prediction by CSI:FingerID on CASMI 2022 benchmark dataset. The best result in each of the testing conditions is indicated in bold font.

Method	Formula Unknown				Formula Predicted				Formula Known				
	Random	DNN	CNN	RNN	DNN	CNN	RNN	CSI:FingerID	Random	DNN	CNN	RNN	CSI:FingerID
Top-*1*	4%	8%	25%	**32%**	12%	23%	**32%**	8%	10%	31%	44%	**54%**	14%
Top-*3*	10%	18%	35%	**43%**	23%	30%	**36%**	13%	22%	44%	53%	**56%**	22%
Top-*5*	16%	25%	38%	**47%**	30%	35%	**41%**	15%	28%	50%	58%	**62%**	25%
Top-*10*	25%	31%	**51%**	**51%**	41%	**49%**	47%	18%	42%	56%	66%	**67%**	28%

## Data Availability

The raw data supporting the conclusions of this article will be made available by the authors on request.

## Supplementary Materials

The following supporting information can be downloaded at https://www.mdpi.com/article/10.3390/metabo15020132/s1. S1: Feature Selection Models; S2: Hyperparameters of Deep Learning Models for Molecular Fingerprint Prediction; Table S1: Top-1 ranking accuracy of candidates for the challenges in CASMI 2016, CASMI 2017, and CASMI 2022 benchmark datasets based on formula prediction by SIRIUS; Table S2: Top-k ranking results for a subset of the challenges in the CASMI 2016 dataset whose MS/MS spectra were acquired in the positive ionization mode; Table S3: Top-k ranking results for a subset of the challenges in the CASMI 2016 dataset whose MS/MS spectra were acquired in the negative ionization mode; Table S4: Top-k ranking results for a subset of the challenges in the CASMI 2017 dataset whose MS/MS spectra were acquired in the positive ionization mode; Table S5: Top-k ranking results for a subset of the challenges in the CASMI 2017 dataset whose MS/MS spectra were acquired in the negative ionization mode; Table S6: Top-k ranking results for a subset of the challenges in the CASMI 2022 dataset whose MS/MS spectra were acquired in the positive ionization mode; Table S7: Top-k ranking results for a subset of the challenges in the CASMI 2022 dataset whose MS/MS spectra were acquired in the negative ionization mode.
