# Supplementary material for: Deep Learning-Based Molecular Fingerprint Prediction for Metabolite Annotation
Source: Metabolites. 2025 Feb 14;15(2):132. doi: 10.3390/metabo15020132 (PMC11857613; doi:10.3390/metabo15020132)
Supplement: Supplementary file 1 [file metabolites-15-00132-s001.zip › metabolites-3432153-supplementary.pdf]

Supplementary Materials

S1. Feature Selection Models

- The Multi-Layer Perceptron (MLP) model used for *m*/*z* bin selection contains three ReLU-activated layers (256, 128, 64 nodes) and a sigmoid-activated dense layer. The learning rate is set to 0.001.
- The Convolutional Neural Network (CNN) model used for fingerprint selection contains four ReLU-activated 1D convolutional layers (32, 64, 128, and 256 nodes respectively), a dropout layer, a data flattening layer, three LeakyReLU-activated dense layers (256, 512 and 1,024 nodes), and a sigmoid-activated dense layer (4,606 nodes). The model learning rate is set to 0.0005.

S2. Hyperparameters of Deep Learning Models for Molecular Fingerprint Prediction

We considered the following deep learning models:

a) Deep Neural Network (DNN): The input vectors first go through a data flattening process, ensuring the data are in the correct format for the fully connected layers. This is followed by three fully connected deep learning layers with 1,024, 2,048, and 4,096 nodes, respectively, designed to extract and learn increasingly complex features. Each deep learning layer is followed by a LeakyReLU activation function to prevent the vanishing gradient problem and improve learning, as well as a dropout layer to reduce overfitting by randomly deactivating neurons during training. The model ends with a sigmoid-activated dense layer consisting of 4,606 nodes, producing class probabilities. The learning rate is set to 0.0005. The model uses the Adam optimizer, which combines adaptive learning rates and momentum to enhance training efficiency and performance.

b) CNN: The input vectors go through four ReLU-activated 1D convolutional layers with 32, 64, 128, and 256 nodes, respectively. Each convolutional layer has a kernel size of 3 and includes L2 kernel regularization (set to 0.001) to prevent overfitting by penalizing large weights. These layers are followed by max-pooling layers to reduce dimensionality while retaining the most important features. After the convolutional layers, a dropout layer with a rate of 0.1 is applied to further mitigate overfitting. The data then pass through a flattening layer and three LeakyReLU-activated dense layers with 256, 512, and 1,024 nodes, respectively, enabling robust feature extraction. The model ends with a sigmoid-activated dense layer containing 4,606 nodes. The learning rate is set to 0.0005. The Adam optimizer is used to achieve efficient optimization. This architecture is optimized for extracting local patterns from the input data.

c) Recurrent Neural Network in Long Short-Term Memory Architecture (RNN/LSTM): The input vectors first go through three LSTM layers with 64, 128, and 256 nodes, each with an L2 kernel regularizer set to 0.001 to prevent overfitting. Dropout layers are applied after each LSTM layer to enhance regularization by reducing the risk of co-adaptation of neurons during training. These LSTM layers are designed to capture sequential and contextual relationships in the data. Following the LSTM layers, the data are passed through a fully connected dense layer with 512 nodes, activated by a LeakyReLU function, and are equipped with an L2 kernel regularizer set to 0.001. A dropout layer is applied after this dense layer for additional regularization. The model ends with a sigmoid-activated dense layer consisting of 4,606 nodes, providing the final probability outputs. The learning rate is set to 0.0005. The model uses the Adam optimizer for training. This architecture is particularly suitable for tasks that involve sequential or temporal dependencies in the input data.

Table S1. Top-1 ranking accuracy of candidates for the challenges in the CASMI 2016, CASMI 2017 and CASMI 2022 benchmark datasets based on formula prediction by SIRIUS.

	CASMI 2016	CASMI 2017	CASMI 2022
Positive	81%	17%	60%
Negative	60%	29%	43%

Table S2. Top-*k* ranking results for a subset of the challenges in the CASMI 2016 dataset whose MS/MS spectra were acquired in the positive ionization mode. The best result in each of the testing conditions is indicated in bold font.

Method	Formula Unknown				Formula Predicted				Formula Known				
	Random	DNN	CNN	RNN	DNN	CNN	RNN	CSI:FingerID	Random	DNN	CNN	RNN	CSI:FingerID
**Top 1**	1%	4%	22%	**26%**	28%	23%	25%	**39%**	15%	43%	**46%**	**46%**	43%
**Top 3**	8%	13%	28%	**30%**	46%	33%	31%	**56%**	24%	58%	57%	57%	**61%**
**Top 5**	14%	20%	34%	**35%**	51%	37%	25%	**58%**	30%	62%	64%	62%	**66%**
**Top 10**	29%	37%	**46%**	**46%**	59%	52%	50%	**63%**	35%	70%	**74%**	**74%**	72%

Table S3. Top-*k* ranking results for a subset of the challenges in the CASMI 2016 dataset whose MS/MS spectra were acquired in the negative ionization mode. The best result in each of the testing conditions is indicated in bold font.

Method	Formula Unknown				Formula Predicted				Formula Known				
	Random	DNN	CNN	RNN	DNN	CNN	RNN	CSI:FingerID	Random	DNN	CNN	RNN	CSI:FingerID
**Top 1**	3%	19%	25%	**28%**	26%	28%	30%	**60%**	13%	40%	46%	48%	**73%**
**Top 3**	11%	28%	31%	**35%**	43%	38%	36%	**63%**	32%	63%	64%	67%	**80%**
**Top 5**	18%	37%	35%	**38%**	53%	44%	47%	**63%**	36%	69%	72%	75%	**80%**
**Top 10**	25%	44%	44%	**49%**	**69%**	56%	57%	63%	41%	78%	81%	**85%**	80%

Table S4. Top-*k* ranking results for a subset of the challenges in the CASMI 2017 dataset whose MS/MS spectra were acquired in the positive ionization mode. The best result in each of the testing conditions is indicated in bold font.

Method	Formula Unknown				Formula Predicted				Formula Known				
	Random	DNN	CNN	RNN	DNN	CNN	RNN	CSI:FingerID	Random	DNN	CNN	RNN	CSI:FingerID
**Top 1**	4%	6%	18%	**33%**	6%	**46%**	30%	9%	7%	18%	**49%**	36%	44%
**Top 3**	13%	9%	23%	**33%**	11%	**54%**	31%	14%	17%	25%	**58%**	42%	57%
**Top 5**	16%	13%	26%	**35%**	14%	**57%**	33%	14%	24%	33%	59%	45%	**62%**
**Top 10**	26%	22%	30%	**40%**	24%	**61%**	39%	15%	38%	40%	62%	50%	**65%**

Table S5. Top-*k* ranking results for a subset of the challenges in the CASMI 2017 dataset whose MS/MS spectra were acquired in the negative ionization mode. The best result in each of the testing conditions is indicated in bold font.

Method	Formula Unknown				Formula Predicted				Formula Known				
	Random	DNN	CNN	RNN	DNN	CNN	RNN	CSI:FingerID	Random	DNN	CNN	RNN	CSI:FingerID
**Top 1**	2%	10%	14%	**19%**	13%	15%	19%	**23%**	5%	22%	22%	**28%**	27%
**Top 3**	9%	13%	**23%**	22%	20%	27%	24%	**29%**	15%	30%	29%	**35%**	34%
**Top 5**	18%	13%	**27%**	24%	26%	**33%**	27%	31%	26%	41%	40%	**43%**	38%
**Top 10**	30%	20%	31%	**32%**	31%	**37%**	34%	33%	34%	50%	49%	**55%**	43%

Table S6. Top-*k* ranking results for a subset of the challenges in the CASMI 2022 dataset whose MS/MS spectra were acquired in the positive ionization mode. The best result in each of the testing conditions is indicated in bold font.

Method	Formula Unknown				Formula Predicted				Formula Known				
	Random	DNN	CNN	RNN	DNN	CNN	RNN	CSI:FingerID	Random	DNN	CNN	RNN	CSI:FingerID
**Top 1**	3%	6%	30%	**35%**	11%	27%	**36%**	13%	9%	27%	47%	**56%**	18%
**Top 3**	8%	19%	37%	**48%**	28%	32%	**40%**	21%	21%	43%	54%	**60%**	28%
**Top 5**	15%	25%	39%	**50%**	33%	36%	**43%**	23%	28%	49%	56%	**62%**	31%
**Top 10**	23%	32%	51%	**55%**	44%	50%	**51%**	27%	43%	54%	67%	**70%**	34%

Table S7. Top-*k* ranking results for a subset of the challenges in the CASMI 2022 dataset whose MS/MS spectra were acquired in the negative ionization mode. The best result in each of the testing conditions is indicated in bold font.

**Method**	**Formula Unknown**				**Formula Predicted**				**Formula Known**				
	Random	DNN	CNN	RNN	DNN	CNN	RNN	CSI:FingerID	Random	DNN	CNN	RNN	CSI:FingerID
**Top 1**	6%	15%	13%	**23%**	13%	10%	**21%**	1%	15%	41%	36%	**46%**	7%
**Top 3**	12%	18%	**28%**	**28%**	15%	23%	**26%**	2%	25%	46%	**51%**	49%	13%
**Top 5**	18%	26%	**36%**	**36%**	23%	**33%**	**33%**	3%	30%	54%	**64%**	62%	16%
**Top 10**	34%	31%	**51%**	41%	31%	**46%**	38%	4%	41%	59%	**64%**	62%	20%
